# Neuronavigation‑guided rTMS of the facial motor cortex for atypical facial pain: Two case reports

**DOI:** 10.1016/j.cnp.2026.01.001

**Published:** 2026-01-10

**Authors:** Dongsheng Xiao, Wei Tao, Yongjie Li, Matilde Balbi, Yuqing Zhang

**Affiliations:** a Queensland Brain Institute, The University of Queensland, Brisbane, QLD 4072, Australia; b Beijing Functional Neurosurgery Institute, Xuanwu Hospital, Capital Medical University, Beijing 100053, China; c Department of neurosurgery, South China Hospital, Medical School, Shenzhen University, Shenzhen, 518116, P. R. China; d Neuromedicine Center, University of Hong Kong-Shenzhen Hospital

**Keywords:** Repetitive transcranial magnetic stimulation, Neuronavigation, Facial motor cortex, Atypical facial pain, Motor cortex stimulation

## Abstract

**Objective:**

Evaluate safety and analgesic effect of neuronavigation-guided 10 Hz rTMS precisely targeting facial M1 in atypical facial pain (AFP).

**Methods:**

Two adults with refractory AFP received 10 daily sessions (2,500 pulses/day) of 10 Hz rTMS at 110 % resting motor threshold. Targeting used MRI/CT-integrated neuronavigation to the facial M1. Pain severity (Barrow Neurological Institute [BNI] scale I–V) and adverse events were recorded through treatment and short follow-up.

**Results:**

Both completed treatment without serious adverse events. Case 1 improved from BNI V at baseline to II at end of treatment, persisting BNI III for 15 days. Case 2 improved from BNI IV to I, with BNI III for 2 days after therapy; both reported better sleep and mood.

**Conclusions:**

Neuronavigation-guided 10 Hz rTMS of facial M1 was well tolerated and associated with rapid, clinically meaningful analgesia in two AFP cases.

**Significance:**

Pilot data support sham-controlled studies to define dose–response and durability, identify responders, and formalize navigated rTMS as a physiological screen to select candidates and targets for implanted brain stimulations.

## Highlights

• Neuronavigation precisely targeted the facial primary motor cortex for stimulation.
• Ten 10 Hz sessions at 110 percent motor threshold produced rapid pain relief.
• Pain scores improved markedly, benefits lasted days, with no serious adverse events.

## 1 Introduction

Atypical facial pain (AFP) is a functional pain syndrome characterized by persistent, often burning or constricting facial pain that does not conform to trigeminal divisions and often includes dysesthesia (Moon, 2010). Symptoms may follow dental or surgical interventions, including trigeminal operations, and tend to be refractory to medication. Standard treatments such as microvascular decompression benefit classical trigeminal neuralgia but are usually ineffective for AFP (Moon, 2010, Koopman et al., 2009).

Repetitive transcranial magnetic stimulation (rTMS) modulates cortical excitability and synaptic plasticity, enabling therapeutic effects across neurological and psychiatric disorders (Barker, 1985, Wassermann and Lisanby, 2001). Analgesic benefits have been most consistently reported with high‑frequency (such as 10  Hz) stimulation of M1, likely via descending inhibitory pathways and network‑level rebalancing of thalamo‑cortical and salience systems (Teng and Mekhail, 2003, Leo and Latif, 2007, Yoo et al., 2006). Targeting accuracy is critical because the M1 facial representation is small and anatomically variable. Neuronavigation integrates the patient’s MRI/CT with live optical tracking of both head and coil, providing on-screen visualization of coil center, orientation, and coil-to-cortex distance; this minimizes targeting error and enables reproducible placement over facial M1.

We report two patients with AFP treated with neuronavigation‑guided 10  Hz rTMS over the M1 facial area and detail targeting workflow, dosing, safety, and outcomes.

## 2 Materials and methods

### 2.1 Study design

Prospective two‑case interventional series at a tertiary neuromodulation center. Patients provided written informed consent for rTMS and for publication of de‑identified data. Screening and consent were documented with institutional forms; procedures adhered to rTMS standard operating procedures (SOPs), including pre‑session checks and post‑session device cooling.

### 2.2 Participants and baseline assessment

Two adults with chronic AFP for 17 and 29 years following trigeminal interventions and suboptimal medication control were enrolled. Both had persistent left facial numbness; Case 2 additionally had perioral clonus. Both were not responsive to common analgesics/painkillers. During the rTMS course, both continued pregabalin and clonazepam at stable doses, and no other concurrent therapies (pharmacological or non‑pharmacological) were introduced. Neuroimaging showed only postoperative changes. Baseline pain severity used the BNI facial pain scale (I–V). Table 1 summarizes demographics and prior procedures.

**Table 1:** Baseline characteristics and surgical histories of the two cases.

Case	Age/Sex	Pain duration (years)	Prior procedures	Pain description	Baseline BNI
#1	63/F	17	Frontal meningioma resection; trigeminal MVD; repeat MVD + partial section of trigeminal sensory root	Continuous constricting pain around left oral commissure and zygoma	V
#2	53/F	29	Nerve blocks; radiosurgery; stellate ganglion block; left MVD + trigeminal root combing	Continuous burning pain in left perioral/buccal regions	IV

### 2.3 Safety screening and consent

Before stimulation, participants completed an adult TMS safety checklist (implants, seizure history, medications), removed ferromagnetic items, and provided written informed consent covering common transient effects (headache, scalp twitching, tinnitus) and the rare risk of seizure. Patients were comfortably seated, their heads stabilized and instructed to wear the ear plugs provided.

### 2.4 Imaging and neuronavigation

Participant’s thin‑slice CT (Case 1) and MRI (Case 2) were reconstructed into a 3D head model. Neuronavigation (Brainsight) co‑registered head and imaging to visualize live coil placement relative to cortical gyri. The hand motor hotspot was localized first, and the coil was then shifted inferior‑laterally to target the facial motor representation on the precentral gyrus.

#### 2.4.1 Facial motor cortex targeting workflow

The facial representation of M1 is small, variable and lies near the inferior precentral gyrus. We combined individual structural imaging, neuronavigation, anatomical landmarks (central sulcus and the Omega sign (inverted‑omega “hand knob”)), and functional TMS‑EMG mapping to ensure reproducible targeting.

**Fig. 1:**
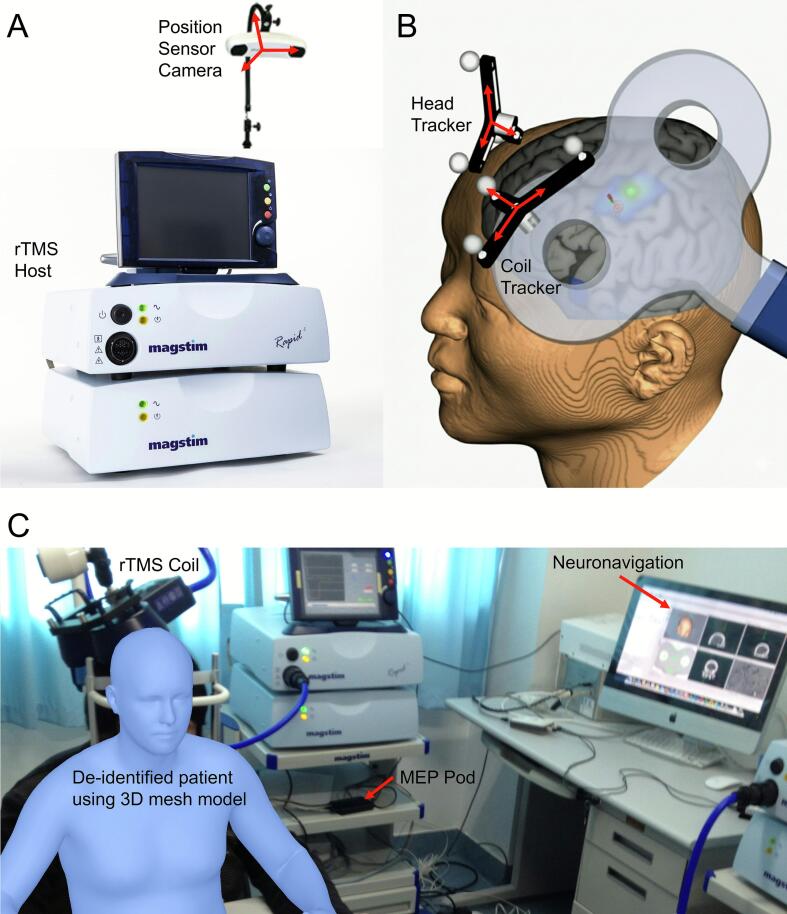
Neuronavigation setup and registration for rTMS. A&B. Hardware layout and optical neuronavigation registration. A coil tracker (rigid body with reflective spheres) is firmly attached to the coil, so its degrees of freedom pose are tracked continuously by the optical camera. Coil and stylus are calibrated before every session. A head tracker is secured to the patient’s forehead. Registration proceeds by touching standard fiducials (nasion and bilateral preauricular points) and then refining with dense scalp surface points. Registration is accepted when root‑mean‑square (RMS) error ≤ 2–3  mm. During targeting, the navigation display shows the live coil vector/plane over the cortical surface and the preplanned facial M1 target. The coil is oriented tangentially to the scalp, typically ∼ 45° to the midline to induce a posterior‑anterior current, while coil‑to‑cortex distance and approach angle are verified. C. Clinical workstation. A Magstim Rapid^2^ stimulator with a figure‑of‑eight coil mounted on a counterbalanced arm. De‑identified patient (3D mesh model (Hu et al., 2022)) seated with head stabilization and ear protection. A navigation workstation (Brainsight) running multiplanar views was used to confirm the central sulcus and hand knob (inverted Ω) landmarks and to monitor coil alignment in real time. Final steps include saving target coordinates and coil pose, capturing target and coil plane, and reverifying registration and coil calibration at each visit to ensure reproducible dosing.
3) Anatomical localization (Fig. 2**A, B**): central sulcus, hand knob, and facial M1. Identify the central sulcus on axial and sagittal views and localize the ‘hand knob’ (inverted‑omega sign) on the precentral gyrus. This defines the hand area. The facial M1 lies inferior–lateral to this site along the precentral gyrus; the upper‑face area is the direct neighbor to the hand area. Mark a provisional facial-area target within the navigation system (saved 3D target and coil pose) and project it to the scalp with the tracked stylus for a skin mark; coil placement is then aligned using real-time navigation.
4) EMG setup. Apply bipolar EMG electrodes to the contralateral abductor pollicis brevis (APB) to identify the hand hotspot and determine resting motor threshold (RMT). Use a forehead/mastoid ground, sampling at 3  kHz with 10–1000  Hz band‑pass and line‑noise notch filtering.
5) Functional mapping with single‑pulse TMS (Fig. 2C). With the figure‑of‑eight coil making contact tangential to the scalp (∼45° from midline to induce a posterior‑anterior current), localize the APB hotspot and determine RMT using the standard ≥ 50  µV in ≥ 5/10 criterion at rest (Rossi et al., 2021). From the APB hotspot, step 5–15  mm inferior–lateral along the precentral gyrus, adjust coil pitch/yaw to remain tangential to the scalp and deliver single pulses at 110–130 % of APB RMT, a supra-threshold range chosen to reliably elicit corticobulbar facial MEPs (thresholds are typically higher than for hand) while remaining within single-pulse safety guidance, while monitoring contralateral orbicularis oris/mentalis EMG for short‑latency MEPs (Rossi et al., 2021). Iterate position/orientation to maximize MEP amplitude and stability. Avoid inadvertent trigeminal nerve or scalp‑muscle activation (palpation/visual inspection).

1) Imaging acquisition and 3D reconstruction. Acquire MRI with a 3D T1‑weighted structural sequence (isotropic voxels) or thin‑slice CT. Export Digital Imaging and Communications in Medicine (DICOM), import into the neuronavigation system, reconstruct the head surface and cortex, and set anatomical orientation lines.
2) Neuronavigation setup and registration (Fig. 1). Calibrate the tracking camera, stylus and coil. Perform head‑to‑image registration using fiducials (nasion and bilateral preauricular points) followed by dense surface‑point refinement over the forehead/temples. Accept registration when RMS error is ≤ 2–3  mm.

**Fig. 2:**
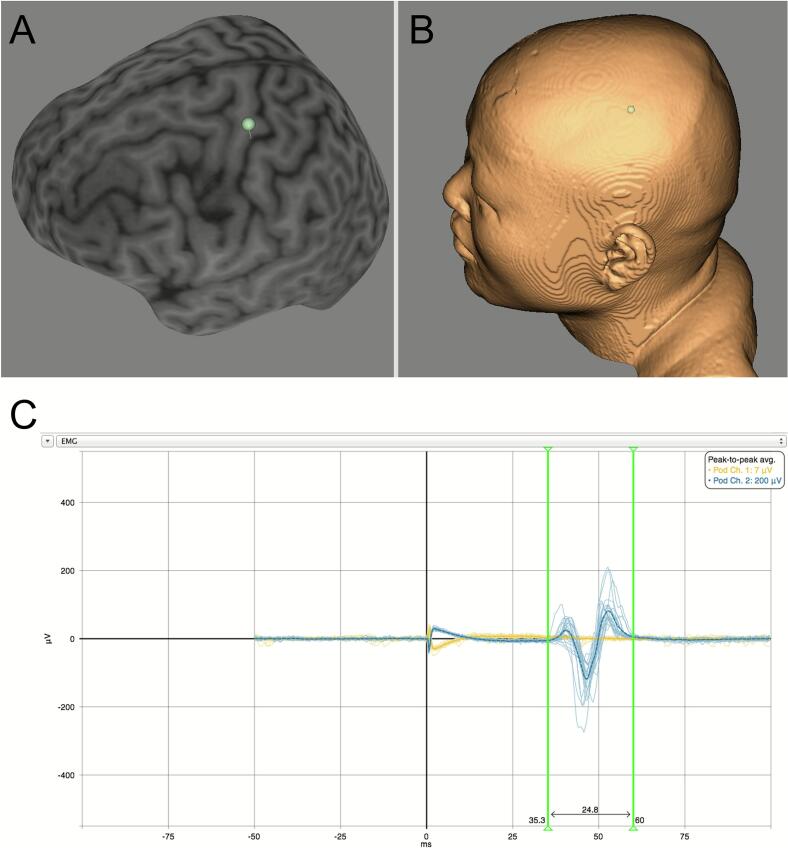
**Anatomical and functional localization of the motor cortex with neuronavigation and TMS-EMG confirmation. A.** Curvilinear cortical surface reconstruction (Brainsight) showing the precentral gyrus; the green sphere marks the navigated target positioned to the hand knob (inverted-Ω) on the precentral gyrus. **B.** Coregistered scalp surface displaying the projected coil placement point (green marker) after fiducial-based head-to-image registration (nasion and bilateral preauricular points with surface-point refinement. **C.** Representative single-pulse TMS–evoked EMG recorded simultaneously from a contralateral hand muscle (blue) and abductor pollicis brevis (APB) control channel (yellow). The black vertical line (0 ms) indicates stimulus onset. (For interpretation of the references to colour in this figure legend, the reader is referred to the web version of this article.)
6) Final target confirmation and documentation. Save the final target coordinates and coil pose in the navigation system, capture the target and coil plane, and record coil‑to‑cortex distance for consistent stimulation targets across sessions. Coil-to-cortex distance was obtained from the neuronavigation readout as the shortest line-of-normal from the tracked coil underside to the cortical target (coil → scalp live + scalp → cortex from CT/MRI), recorded each session for ± 2 mm reproducibility. Lock the coil in position for treatment. Reverify head registration and coil calibration at each visit and recheck RMT periodically to maintain dose fidelity.

### 2.5 Stimulation hardware and coil

A Magstim Rapid^2^ stimulator with a figure‑of‑eight coil delivered focal stimulation. Coil orientation was adjusted to maximize tangential induced current over the M1 facial hotspot with head stabilization and earplugs per SOP.

### 2.6 Resting motor threshold (RMT)

RMT was determined at the contralateral abductor pollicis brevis hotspot using standard procedures (Rossi et al., 2021) (minimum output generating ≥ 50  µV MEPs in ≥ 5/10 trials at rest). Treatment intensity was set to 110 % RMT and rechecked as needed.

### 2.7 rTMS parameters and schedule

rTMS was delivered at 10  Hz, in 10‑s trains with 50‑s inter‑train intervals (1:5 duty cycle) for 25 min, totaling 2500 pulses per session, 5  days/week for 2  weeks (10 sessions). Parameters balanced efficacy and safety according to institutional guidance and prior literature.

### 2.8 Outcomes and follow‑up

Primary outcome: BNI facial pain score change from baseline to end‑of‑treatment and during near‑term follow‑up. Adverse events were actively queried at each session (seizure, syncope, headache, scalp discomfort/twitching, dizziness, tinnitus, mood/sleep changes). If an adverse event occurred, stimulation was paused and documented; mild/transient events were managed (minor dose/interval/position adjustments) and resumed after resolution, whereas moderate/severe or neurological events led to session termination, SOP-based clinical evaluation, and deferral of further sessions.

## 3 Results

### 3.1 Safety and tolerability

Both cases completed 10 sessions without seizures, syncope, or other serious adverse events. Both reported improved sleep and mood in parallel with analgesia.

### 3.2 Case 1

**Fig. 3:**
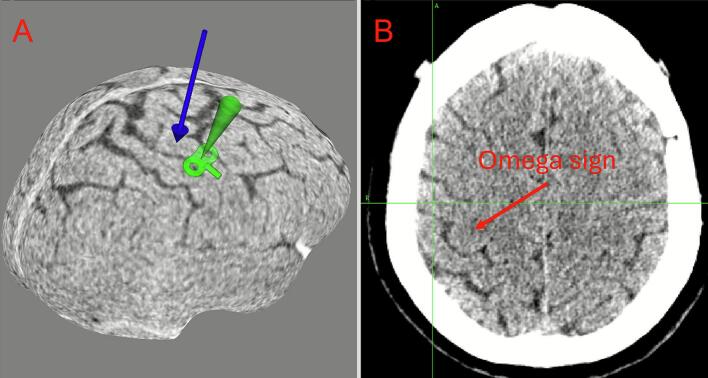
**Case 1 neuronavigation target. A.** Thin‑slice CT 3D reconstruction with target overlay near the inferior precentral gyrus. The blue arrow marks the M1 hand area; the green shading denotes the stimulation field of the figure‑of‑eight coil, targeting the facial motor area. **B.** An axial image of the stimulation plane. (For interpretation of the references to colour in this figure legend, the reader is referred to the web version of this article.)

A 63‑year‑old woman with 17  years of facial pain had continuous constricting pain around the left oral commissure and zygomatic region (BNI V). Pain relief began after session 5; by session 10 she reached BNI II, and this relief persisted up to 15 days post‑course at approximately BNI III (Fig. 3, Fig. 5, Table 1).

**Fig. 4:**
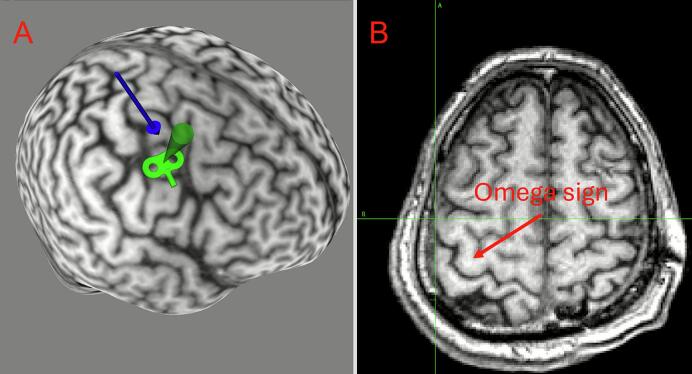
**Case 2 neuronavigation target. A.** MRI 3D reconstruction with target overlay. The blue arrow marks the M1 hand area; the green shading denotes the stimulation field, targeting the facial motor area. **B.** An axial image of the stimulation plane. (For interpretation of the references to colour in this figure legend, the reader is referred to the web version of this article.)

**Fig. 5:**
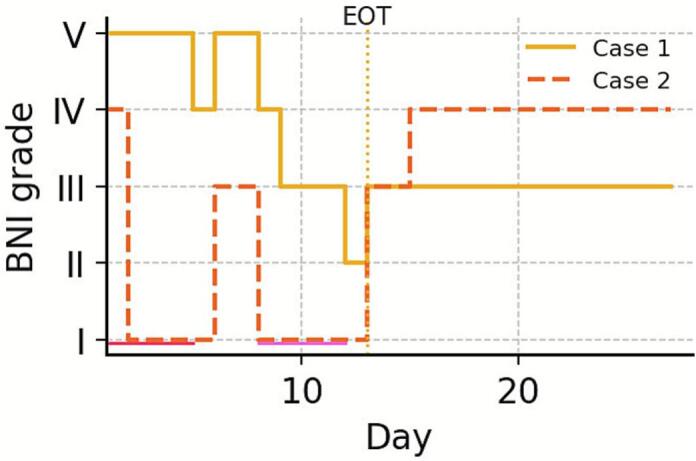
**Daily BNI facial pain grade during treatment and short‑term follow‑up.** Step curves show each participant’s daily Barrow Neurological Institute (BNI) facial pain grade (I = best, V = worst). Day 1 is the first rTMS session; sessions were delivered on Days 1–5 (red line) and Days 8–12 (pink line) (two‑day-break Days 6–7); end of treatment (EOT) = Day 13. All sessions were attended; horizontal segments indicate the grade persists until the next observation. (For interpretation of the references to colour in this figure legend, the reader is referred to the web version of this article.)

### 3.3 Case 2

A 53‑year‑old woman with 29  years of refractory left perioral/buccal burning pain (BNI IV) experienced complete relief lasting > 2 h after session 2 and reached BNI I at end‑of‑course; relief persisted ∼ 2 days post‑course at approximately BNI III (Fig. 4, Fig. 5, Table 1).

## 4 Discussion

This two‑case series suggests that neuronavigation‑guided 10  Hz rTMS targeting the M1 facial area can rapidly reduce AFP symptoms with short‑term persistence of benefit and excellent tolerability. The time‑course of induced analgesia (onset by session 2–5; persistence beyond the last session) is consistent with rTMS‑induced plasticity that outlasts stimulation.

M1 stimulation may recruit descending inhibitory pathways (periaqueductal gray/rostral ventromedial medulla), modulate thalamo‑cortical gating, and rebalance sensorimotor–salience networks implicated in pain processing (Teng and Mekhail, 2003, Leo and Latif, 2007, Yoo et al., 2006). Among candidate cortical sites, M1 has shown superior analgesic effects relative to S1, premotor, or supplementary motor areas (Wassermann and Lisanby, 2001).

Accurate targeting is critical for the small, variable M1 facial representation; neuronavigation enables individualized coil‑to‑cortex alignment, improving fidelity and reproducibility and allowing documentation for maintenance sessions or future invasive trials (e.g., epidural motor cortex stimulation (MCS)) (Hirayama et al., 2006). Navigated rTMS can operate as a pragmatic physiological screen to select candidates and localize effective cortical targets for MCS (Zhang et al., 2017).

Notably, neuronavigation was based on thin‑slice CT in Case 1 and MRI in Case 2. MRI typically provides superior cortical surface definition and gyral/sulcal contrast, which may facilitate more confident anatomical targeting of the small and variable facial M1 representation. Conversely, thin‑slice CT provides highly accurate geometric information for scalp/skull surface reconstruction and can support robust registration. In both cases we accepted registration only when RMS error was ≤ 2–3  mm and documented the target and coil pose to maximize reproducibility across sessions. Given the two‑case design, we cannot attribute the greater clinical response in Case 2 to imaging modality alone; between‑case differences and individual neurophysiological responsiveness may also contribute.

Limitations include the uncontrolled design (n = 2), ordinal outcome (BNI) rather than quantitative scales, and potential placebo effects. Durability, maintenance schedules, and predictors of response require testing in sham‑controlled trials.

## 5 Conclusions

Neuronavigation‑guided 10  Hz rTMS of the M1 facial representation was safe and associated with clinically meaningful, short‑term analgesia in two patients with postoperative AFP after trigeminal procedures. Controlled studies are warranted to establish efficacy, durability, optimal dosing, and patient selection.

## Ethics approval and consent to participate

Procedures complied with institutional policies and the Declaration of Helsinki. According to local policy for case reports/case series, the requirement for formal ethics committee review was waived. Each patient gave written informed consent to rTMS treatment and publication of de‑identified information. Written consent for publication of de‑identified case details and images was obtained from both patients.

## Funding

Dr. Matilde Balbi is supported by the NHMRC Ideas Grant (2022/GNT2020164), Brazil Family Program for Neurology. Dr. Yuqing Zhang is supported by the Wu Jieping Medical Foundation (320.6750.19089-78).

## Declaration of Competing Interest

The authors declare that they have no known competing financial interests or personal relationships that could have appeared to influence the work reported in this paper.
